# Amharic political sentiment analysis using deep learning approaches

**DOI:** 10.1038/s41598-023-45137-9

**Published:** 2023-10-20

**Authors:** Fikirte Alemayehu, Million Meshesha, Jemal Abate

**Affiliations:** 1 Department of Information Science, Haramaya University, Dire Dawa, Ethiopia; 2 School of Information Science, Addis Ababa University, Addis Ababa, Ethiopia; 3 Haramaya, Ethiopia

**Keywords:** Computer science, Information technology

## Abstract

This study delves into the realm of sentiment analysis in the Amharic language, focusing on political sentences extracted from social media platforms in Ethiopia. The research employs deep learning techniques, including Convolutional Neural Networks (CNN), Bidirectional Long Short-Term Memory (Bi-LSTM), and a hybrid model combining CNN with Bi-LSTM to analyze and classify sentiments. The hybrid CNN-Bi-LSTM model emerges as the top performer, achieving an impressive accuracy of 91.60%. While these results mark a significant milestone, challenges persist, such as the need for a more extensive and diverse dataset and the identification of nuanced sentiments like sarcasm and figurative speech. The study underscores the importance of transitioning from binary sentiment analysis to a multi-class classification approach, enabling a finer-grained understanding of sentiments. Moreover, the establishment of a standardized corpus for Amharic sentiment analysis emerges as a critical endeavor with broad applicability beyond politics, spanning domains like agriculture, industry, tourism, sports, entertainment, and satisfaction analysis. The exploration of sarcastic comments in the Amharic language stands out as a promising avenue for future research.

## Introduction

In 2020, over 3.9 billion people worldwide used social media, a 7% increase from January. About 4.9 billion people accessing social media globally as of 2023. An average social media user hops between 6 and 7 platforms every month. While there are many factors contributing to this user growth, the global penetration of smartphones is the most evident one^1^. Some instances of social media interaction include comments, likes, and shares that express people’s opinions. This enormous amount of unstructured data gives data scientists and information scientists the ability to look at social interactions at an unprecedented scale and at a level of detail that has never been imagined previously^2^. Analysis and evaluation of the information are becoming more complicated as the number of people using social networking sites grows. For example, Facebook, Instagram, e-commerce websites, and blogs improve customer satisfaction and the overall shopping experience for the customer by allowing customers to rate or comment on the products they have purchased or are planning to purchase^3^.

Sentiment analysis, also known as Opinion mining, is the study of people’s attitudes and sentiments about products, services, and their attributes^4^. Sentiment analysis holds paramount importance in political discourse, particularly within the Amharic-speaking region of Ethiopia^5^. Instances from global and local political landscapes underscore the impact of sentiment analysis on political reform. For instance, the 2008 election of Barack Obama in the United States showed the role of social media in shaping political sentiment, galvanizing support, and mobilizing voters. Within Ethiopia itself, sentiment analysis has been closely linked to political reform. The Ethiopian political landscape has undergone significant changes in recent years, and social media has helped to voice public opinion and influencing political decisions. Social media sites such as Facebook, Twitter, and YouTube were being used to assist in a country’s political reform process.

Analyzing Amharic political sentiment poses unique challenges due to the diversity and length of content in social media comments. The Amharic language encompasses a rich vocabulary and intricate grammatical structures that can vary across regions and contexts. This linguistic complexity complicates sentiment analysis, necessitating context-aware approaches. Moreover, social media comments are often lengthy and contextually nuanced, making it challenging to accurately capture the intended sentiment^5^.

While previous works have explored sentiment analysis in Amharic, the application of deep learning techniques represents a novel advancement. By leveraging the power of deep learning, this research goes beyond traditional methods to better capture the Amharic political sentiment. The uniqueness lies in its ability to automatically learn complex features from data and adapt to the intricate linguistic and contextual characteristics of Amharic discourse. The general objective of this study is to construct a deep-learning sentimental analysis model for Amharic political sentiment.

## Related works

Sentiment analysis, which involves categorizing sentiments as positive or negative, has been explored across various domains in local contexts. Various researchers have applied machine learning techniques to perform sentiment analysis in domains such as entertainment^6^, aspect-level sentiment classification from social media^7^, and deep learning-based Amharic sentiment classification^8^.

Hassan and Mahmood^9^ employed deep learning for sentiment analysis on short texts using datasets like Stanford Large Movie Review (IMDB) and Stanford Sentiment Treebank. Word2Vec was utilized for word embedding, combining Convolutional Neural Networks (CNN) with recurrent neural networks (RNN). Despite achieving 88.3% and 47.5% accuracy, the hybrid model was deemed suboptimal, suggesting further experimentation with different RNN models.

Ghorbani et al.^10^ introduced an integrated architecture of CNN and Bidirectional Long Short-Term Memory (LSTM) to assess word polarity. Despite initial setbacks, performance improved to 89.02% when Bidirectional LSTM replaced Bidirectional GRU. This study underscores how model compatibility impacts performance. Mohammed and Kora^11^ tackled sentiment analysis for Arabic, a complex and resource-scarce language, creating a dataset of 40,000 annotated tweets. They employed various deep learning models, including CNN and Long Short-Term Memory (LSTM), achieving accuracy rates ranging from 72.14 to 88.71% after data augmentation.

Meena et al.^12^, demonstrate the effectiveness of CNN and LSTM techniques for analyzing Twitter content and categorizing the emotional sentiment regarding monkeypox as positive, negative, or neutral. The effectiveness of combining CNN with Bidirectional LSTM has been explored in multiple languages, showing superior performance when compared to individual models. Noteworthy studies include Shen et al.^13^ for IMDB movie reviews, Zhou et al.^14^ for Chinese product reviews, Alharbi^15^ for Arabic datasets, and Ref.^16^ for Afaan Oromo datasets. Meena et al.^17^, proposes an effective sentiment analysis model using deep learning, particularly the CNN strategy, to evaluate customer sentiment from online product reviews. The findings suggest the potential for using online reviews to inform future product selections. While the study focused on laptops, phones, and televisions, there’s room for extending this approach to different products and languages in future research. Several researchers have endeavored to build sentiment classification models for Amharic. Abraham^6^ applied machine learning to Amharic entertainment texts, achieving 90.9% accuracy using Naïve Bayes. However, challenges remain, such as handling negation and exploring n-grams for improved feature sets. Aspect-level opinion mining is also suggested for further research.

Mulugeta and Philemon^18^ utilized supervised machine learning with Naïve Bayes and Bigram for sentiment analysis in Amharic, presenting an alternative multi-scale approach. Despite limited training data, results were encouraging, leading to the proposal of further research in document-level sentiment analysis. Yeshiwas and Abebe^8^ adopted a deep learning approach for Amharic sentiment analysis, annotating 1600 comments with seven classes. Using CNN and various experiments, they achieved accuracy rates ranging from 40 to 90.1%. These findings laid the foundation for future exploration of Amharic sentiment analysis. Turegn^19^ evaluated the impact of data preprocessing on Amharic sentiment analysis, integrating emojis, and comparing human and automatic annotation. The study found that stemming had no positive impact, emojis provided a negligible improvement, and automatic annotation overlapped significantly with human annotation. The study suggested further exploration of CNN-LSTM and CNN-BiLSTM networks to enhance prediction accuracy.

Mengoni and Santucci^20^, highlights the recent strides in Artificial Intelligence, particularly in Natural Language Processing (NLP), tackling tasks from machine translation to sentiment analysis. While these achievements are notable, challenges persist, including adapting English-based NLP methods to other languages. These studies collectively underline the evolution of Amharic sentiment analysis and its challenges, providing valuable insights for future research. The summary of related research works has been depicted in Table 1 as follows.

**Table 1:** Summary of related works.

S.no.	Study	Objective	Methodology	Results and conclusions
1	Hassan and Mahmood^9^	Apply deep learning for sentiment analysis of short texts	CNN and RNN Word2Vec for word	Accuracy of 88.3% and 47.5% Suggested further experimentation with other RNN models
2	Ghorbani et al.^10^	Develop integrated CNN and LSTM architecture for word polarity identification	CNN and LSTM Glove for word embedding	Replaced bidirectional GRU with LSTM for improved performance (89.02%) Suggested multiclass classification and sarcastic comment identification
3	Mohammed and Kora^11^	Create a deep learning model for Arabic sentiment analysis	CNN, RNN, and RCNN Aravec word embeddings	Accuracy for CNN (72.14–77.60%), LSTM (80.91–81.53%), and RCNN (78.24–78.82%) Suggested data augmentation to enhance LSTM performance
4	Abraham^6^	Apply machine learning algorithms for sentiment analysis of Amharic entertainment texts	Naïve Bayes, J48 Decision Tree, and Maximum Entropy classifiers	Naïve Bayes achieved the highest accuracy (90.9%) Recommended using bi-gram and trigram for the better featsets set and exploaspect-levellevel opinion mining
5	Philemon^18^	Implement supervised machine learning for Amharic sentiment analysis with a multi-scale approach	Naïve Bayes with Bigram	Accuracy of 39.5–44.3% Suggested further work in document-level sentiment analysis
6	Yeshiwas and Abebe^8^	Develop a deep learning model for Amharic sentiment analysis	CNN and Scikit-learn’s Count Vectorizer and TF-IDF	Average accuracy of 40.1–90.1% using CNN with different training/testing splits Suggested using more network layers for improved performance
7	Turegn^19^	Evaluate preprocessing and Emoji effects on Amharic sentiment analysis using LSTM	LSTM with Word2Vec for word embedding	Preprocessing, like stemming, reduced accuracy (75.93% from 82.36%) Emojis improved accuracy by 0.55%. Automatic and human annotations overlapped by 90.67% Suggested collaboration of CNN and LSTM for future work

## Research methodology

### Overview

This study has implemented an experimental research method. Experimental research design is a scientific method of investigation in which one or more independent variables are altered and applied to one or more dependent variables to determine their impact on the latter. In experimental research, experimental setup such as determining how many trials to run and which parameters, weights, methodologies, and datasets to employ.

### Data collection and preparation

#### Data collection

A total of 5000 comments were acquired for this study from different sources that prominently discuss the political environment in Ethiopia. To ensure the correctness and relevance of the collected sentiments, this process was carried out in close collaboration with a linguistic expert. To keep the dataset balanced, an equal distribution of positive and negative comments was maintained. In the process of data acquisition, lexicons employed by prior researchers^7, 21^ were used. The data source of this study was the official social media pages affiliated with Prime Minister Dr. Abiy Ahmed, Fana Broadcasting Corporation (FBC), the Ezema political party’s official Facebook page, and the Prosperity Party’s official Facebook account.

#### Dataset preparation

Once the dataset was collected, a careful process of data organization and cleansing was followed. The goal was to eliminate inconsistencies, and typographical errors, as well as duplicate or inaccurate information that might distort the integrity of the dataset. The data cleaning stage helped to address various forms of noise within the dataset, such as emojis, linguistic inconsistencies, and inaccuracies. Short forms of words were expanded to full forms, stop words were removed, and synonyms were converted into normalized forms during preprocessing.

### Deep learning approaches used

Various deep-learning models exist for sentiment classification. In this study, the selection of deep learning models was contingent on their suitability for Amharic sentiment analysis. During the model selection process criteria that is noted by Refs.^22–24^ were considered. These criteria encompass aspects such as feature extraction proficiency, the preservation of long-term dependencies, mitigation of the vanishing gradient problem, aptitude in comprehending diverse linguistic contexts, as well as models characterized by fewer parameters and faster convergence times.

#### CNN

**Figure 1:**
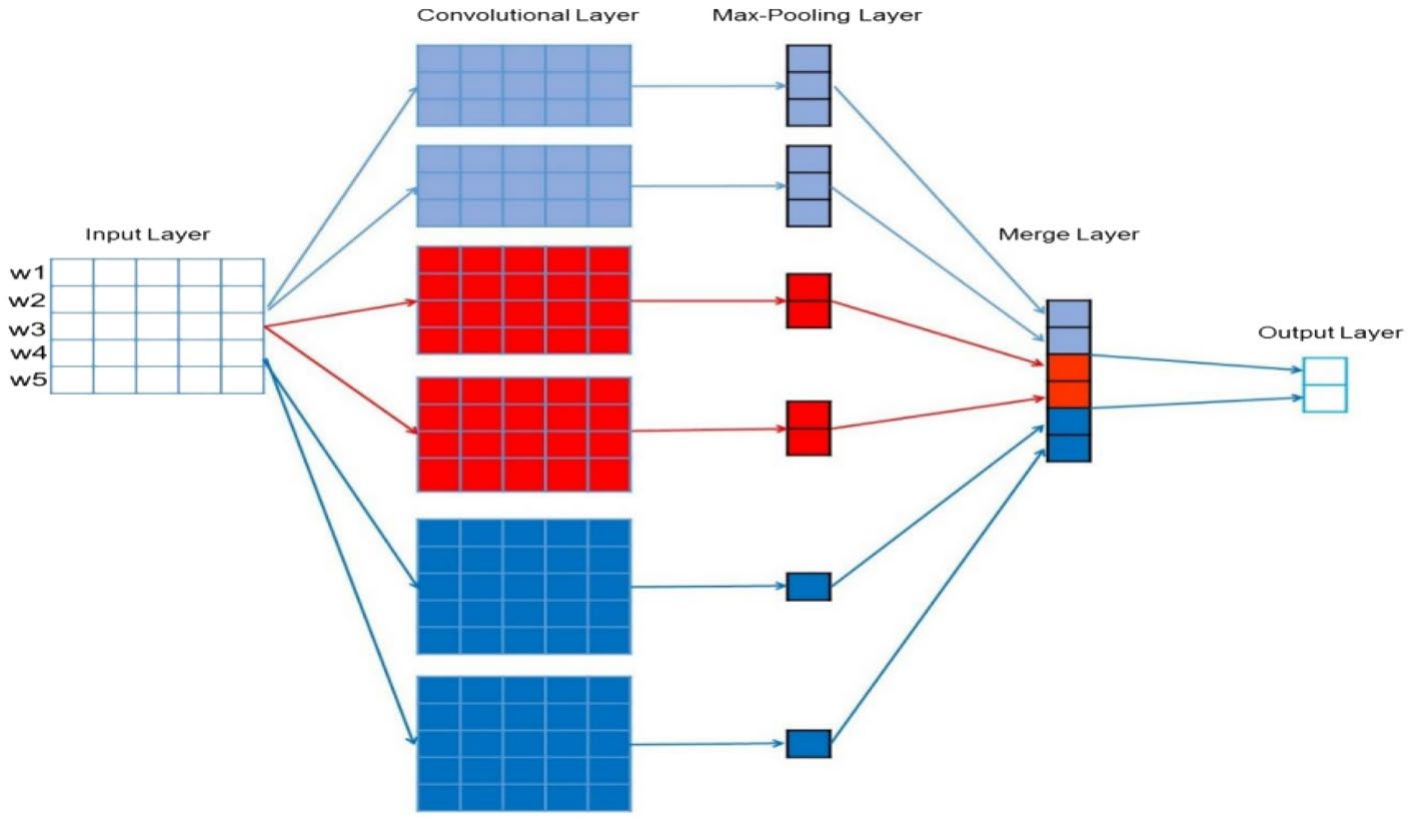
CNN model architecture for text classification^24^.

CNN models use a convolutional layer and pooling layers to extract high-level features. For this research, a 1D CNN for sentiment words, which treats sentiment as a one-dimensional collection of pixels was employed. CNN is used to find hidden connections between words in the nearby region. CNN is recognized for its capability to extract features accurately and minimizing the number of input features. It is built by applying the different steps^24^. First embedded words are fed into the convolutional layer, which selects the features, and then the pooling layer performs dimensionality reduction on the feature extracted on the previous layer after the features are combined then passed into the fully connected layer, where the output is determined based on Sigmoid function that normalizes into the two classes (i.e., positive, and negative). Figure 1 presents the architecture of the CNN model used for text classification.

#### Bidirectional-LSTM

**Figure 2:**
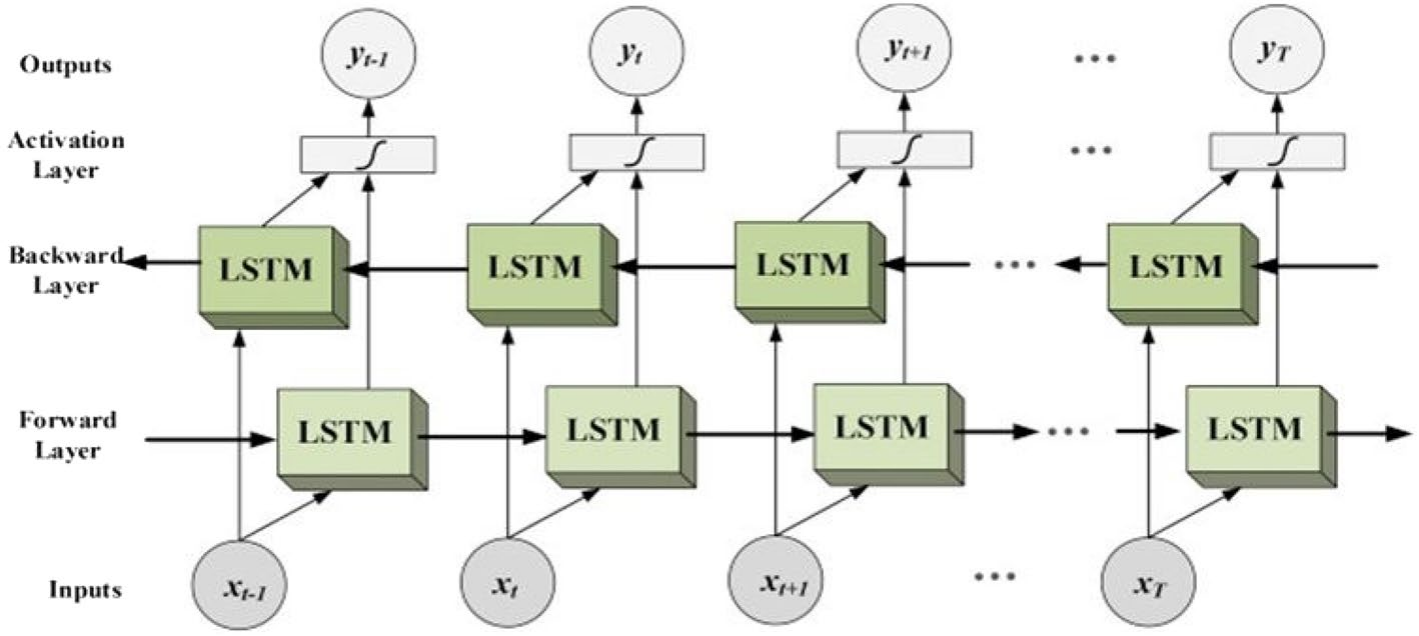
Bidirectional-LSTM^26^.

Long short-term memory networks that are bidirectional can incorporate context information from both past and future inputs^25^. Over long sequences, parts of the gradient vector may exponentially expand or decline, making it challenging for RNN to include long-term dependencies. The LSTM design overcomes the issue of learning long-term dependencies presented by the simple RNN by incorporating a memory cell that can hold a state over a long period. In a way, the Bidirectional-LSTM combines the forward hidden layer with the backward hidden layer (see the Fig. 2), to manipulate both previous and future input.

It can be seen from Fig. 2 that Bi-LSTM can learn in both directions and integrate the pieces of knowledge to make a prediction. The embedded words were used as an input for bidirectional LSTM model and added a BI-LSTM layer using Keras. TensorFlow’s Keras now has a new bidirectional class that can be used to construct bidirectional-LSTM and then fit the model to our data.

#### Gated recurrent unit (GRU)

GRU uses gating units that influence the flow of information within the unit to address the vanishing gradient problem of a regular RNN. Large texts benefit greatly from GRU. GRU like LSTM has gating units that regulate data flow but unlike LSTM there is no need for additional designated memory cells. The update and reset gates are two crucial gates of GRU that decide what information should be passed to the output^27^.

**Figure 3:**
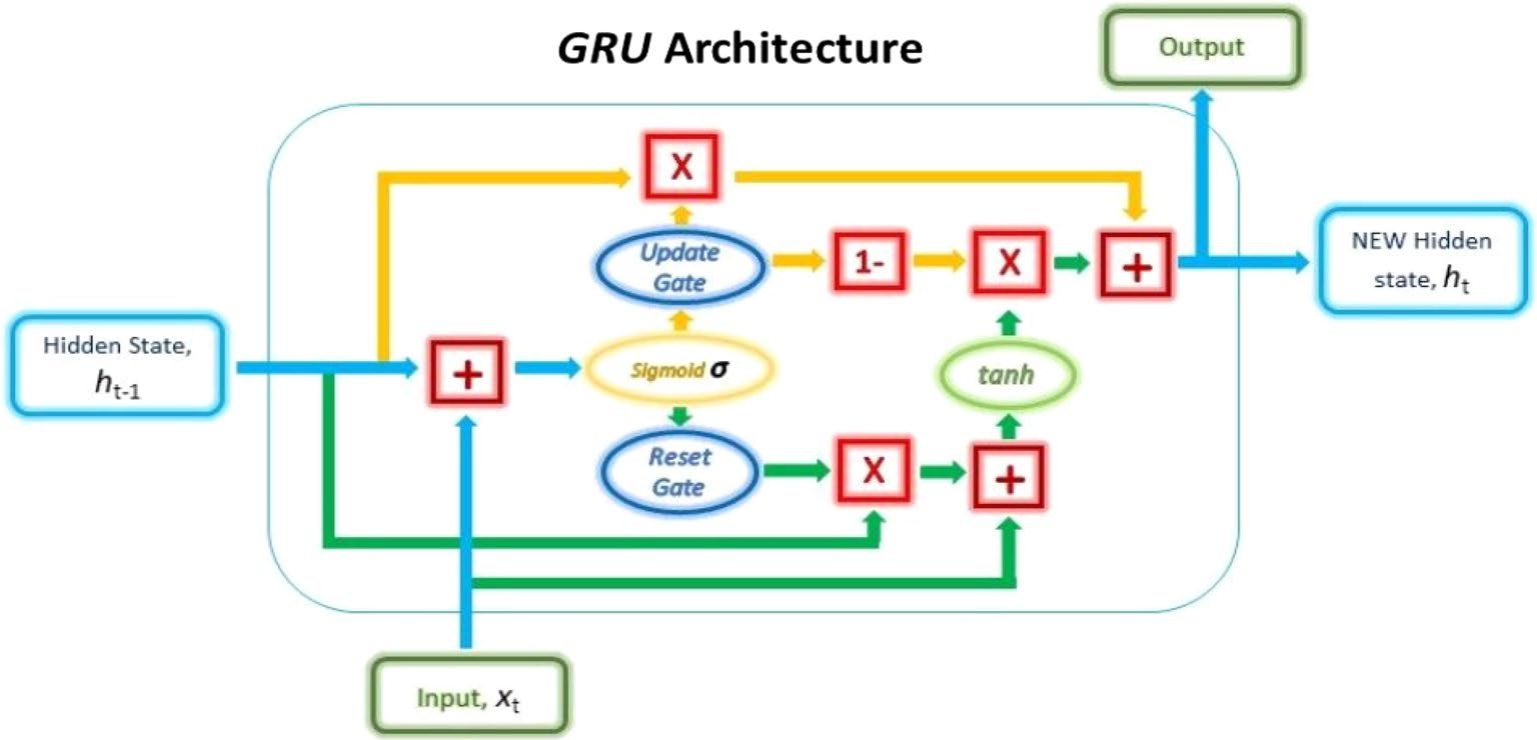
The internal structure of GRU^23^.

The architecture depicted in Fig. 3 shows how GRU uses the two gates for output determination. The reset gate determines whether parts of the prior hidden state should be integrated with the present input to formulate a new hidden state. The update gate oversees deciding just how much of the prior hidden state should be kept and how much of the proposed new hidden state from the Reset gate should be included in the final hidden state. Whenever the Update gate is multiplied with the prior hidden state for the first time, the gate chooses which pieces of the prior hidden state to preserve in memory and dismiss the rest. As a result, whenever it utilizes the reverse of the Update gate to extract the newly proposed hidden state from the Reset gate, it is filling up the required pieces of information^23^.

#### Hybrid CNN-bidirectional-LSTM

**Figure 4:**
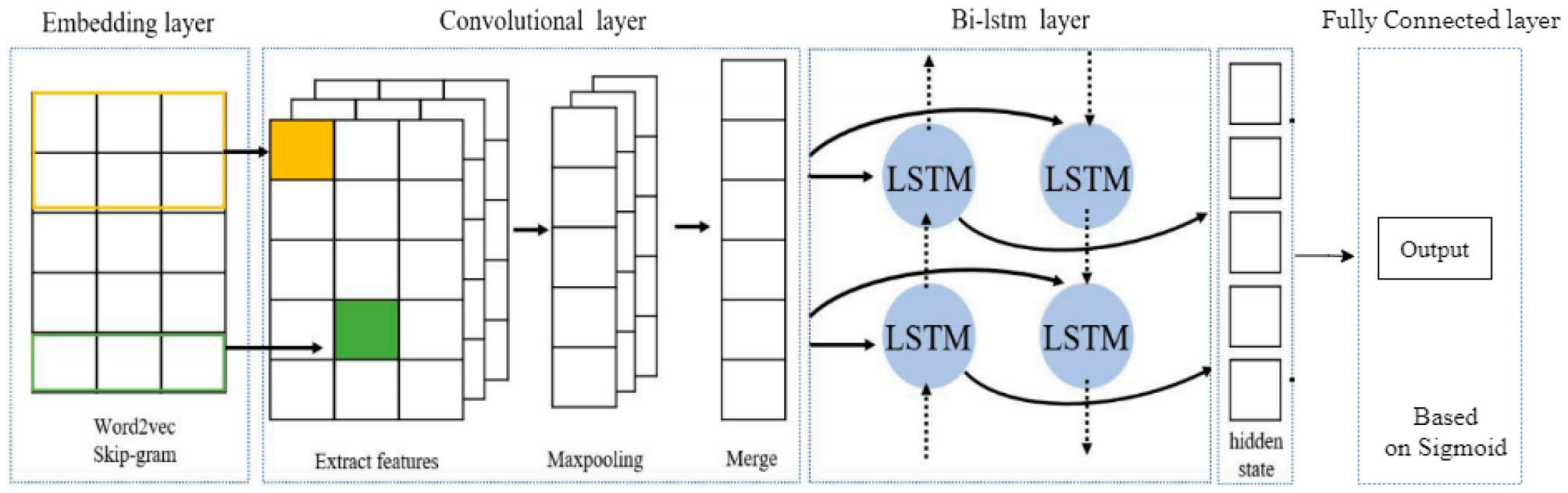
The proposed model architecture^22^.

The strengths of CNN and Bi-directional models are combined in this hybrid technique (see Fig. 4). CNN models use convolutional layers and pooling layers to extract features, whereas Bidirectional-LSTM models preserve long-term dependencies between word sequences^22^. Hence CNN-Bidirectional-LSTM models are more suitable for sentiment classification.

The inputs are preprocessed and embedded before it is passed to CNN. Convolutional layers extract features from different parts of the text and the pooling layer reduces the number of features in the input. Then features obtained from the pooling layer are passed to the Bidirectional-LSTM to extract contextual information. Finally, the last states of the BiLSTM are concatenated and passed into the Sigmoid activation function, which squashes the final value in the range between 0 and 1.

## Proposed architecture and design

**Figure 5:**
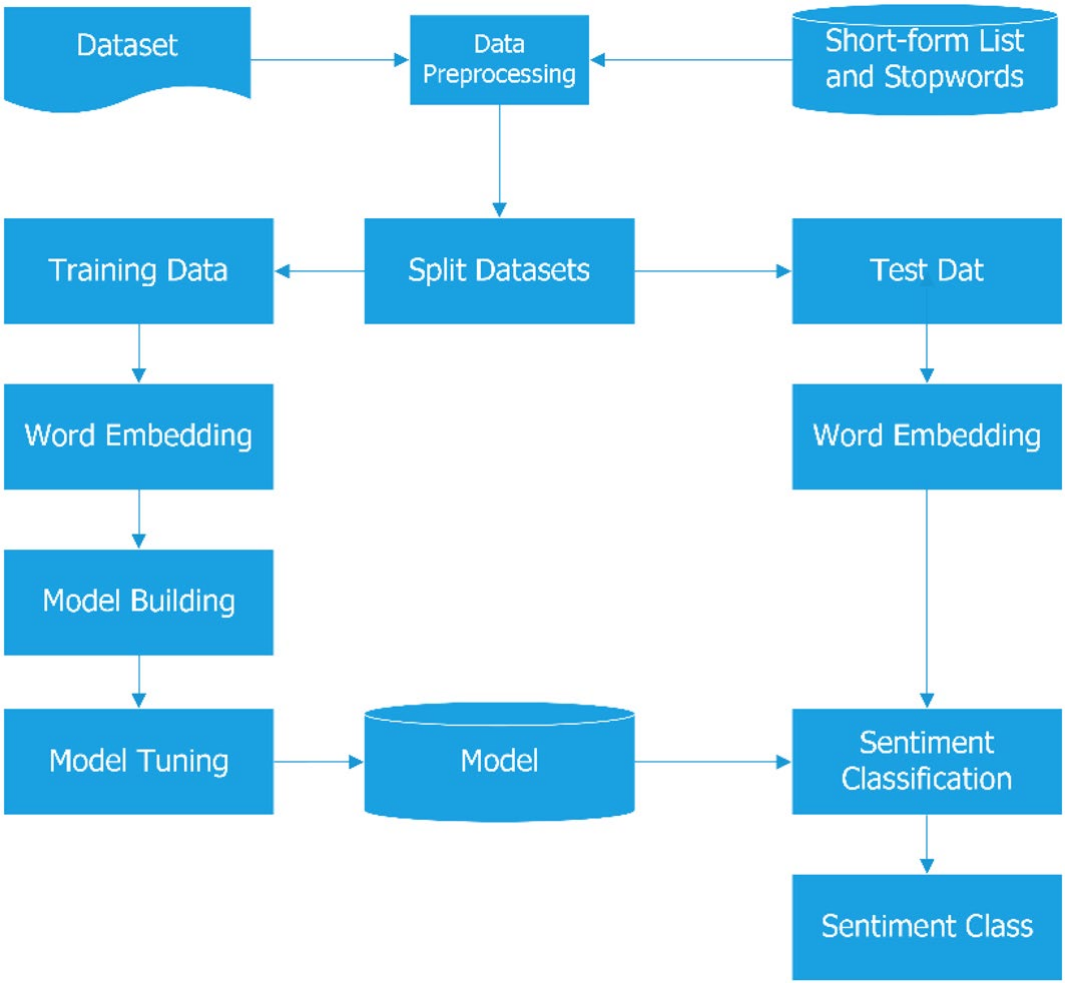
Architecture of sentiment analysis for amharic language using deep learning.

The general Architecture of Amharic sentimental analysis using a deep learning approach is shown in Fig. 5 below.

### Data preprocessing

Data preprocessing is the process of removing distortion from data to make any classification task easier in our case sentiment classification and improve the performance of the model. As a result, it is critical to apply data preprocessing to overcome such issues because the more the data is cleaned the more accurate the deep learning model will be.

**Figure 6:**
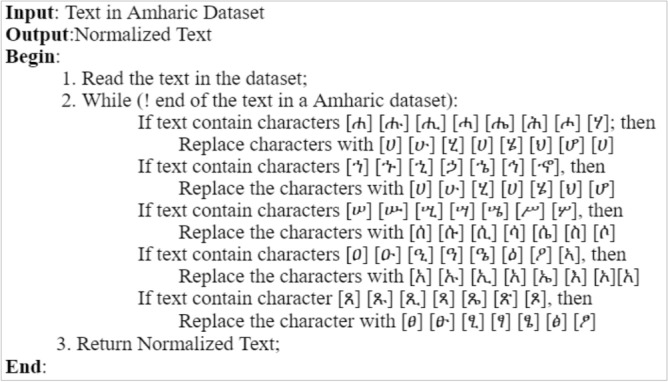
Algorithm for normalization of Amharic character variants.

- *Short-form expansion* In Amharic, there is a lot of short form that need to be expanded to get the full-length word because the researcher is using the word to train our data. Some of the short forms used frequently in writing comments and opinions in Amharic are shown in Table 2 below.
- *Data cleaning* In this stage of preprocessing, eliminate any special characters, symbols, and emojis that aren’t needed. It was started by removing all non-Amharic characters and any special characters shown below in Table 3.
- *Normalization* In Amharic, there are different characters that have the same sound but are written in different forms like ^28^. The description of the algorithm used for transforming text into a single canonical form is depicted in Fig. 6 below.
- *Tokenization* Larger chunks of a text document can be tokenized into a list of sentences, and sentences into a list of words. The list of words identified by the tokenizer function is then used for training and also testing. To be comprehended by the deep learning system, such tokens are also transformed to vector format.
- *Stop-words detection and removal* Stop words must be removed to reduce the dimensionality of the word vector because they have no contribution in determining emotion or sentiment. Some of the most common stop words in Amharic language are  etc.
- *Padding* Deep learning networks expect datasets to have vectors with equal dimensions. However, not all sentences are the same size after preprocessing. To put it another way, some of the sentences are longer or shorter in terms of the word they contain. To make the documents uniform in size, a zero is added pre the sentence or post the sentence of the shorter sentence matrices which is called Padding. Sentences with numerically represented words and a maximum length of a given sentence is used as an input. If the sentence is less than the maximum length post padding is applied which is adding zero at the end of the sentence to make it equal to the maximum length of a sentence which in this research is 20.

**Table 2:**
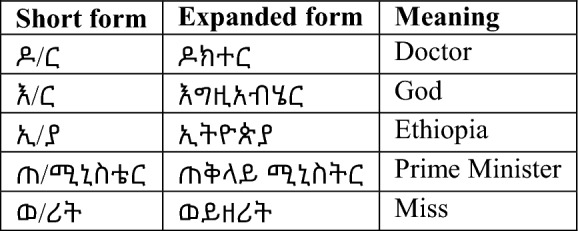
Amharic short forms in writing.

**Table 3:**
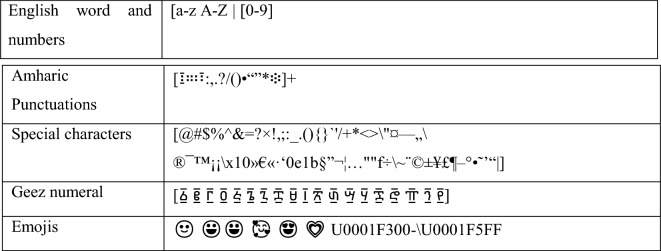
Removed words, numbers, and punctuations.

### Word-embedding

Word-embedding is a feature learning technique in which each word or phrase in the vocabulary is mapped to an N-dimensional real-number vector. The goal of word embedding is to convert all words in the dictionary into a lower-dimensional vector. To build a word representation of the data for the deep learning model the researcher employs Word2Vec as an embedding model. After preprocessing and converting the datasets to a format that can be analyzed, the words in the sentence must be represented as vectors so that Word2Vec can calculate similarity, analogy. The embedding layer converts the input into an $N\times M$ dimensional vector, where N represents the longest sentence in the dataset and M represents the embedding dimension.

## Experimental result

Four experiments were conducted by dividing the preprocessed dataset into three subsets which was 4000 sentences for training, 500 for validation, and another 500 for testing.

### Experimenting using CNN

In the CNN experimentation, we began by inputting the preprocessed data into the CNN layer to facilitate feature extraction. The CNN layer employed 128 filters with 5 kernels and utilized the ReLU activation function. Following this feature extraction step, the data was forwarded to the GlobalMaxPooling1D layer, which downed sample the representation by selecting the maximum value across time, converting the output from 2 to 1D. Subsequently, these values were passed to the fully connected output layer. To maintain output values between 0 and 1 for the binary classification task of negative and positive sentiment, a sigmoid activation function was applied. Binary cross-entropy was chosen as the loss function. During training, the researcher measured accuracy, recall, and precision as performance metrics and conducted training over 10 epochs to optimize the model. The model is assessed on the test dataset once the model is fitted; the result is presented as shown below in Table 4.

**Table 4:** 1st evaluation result for CNN model.

Metrics	Training (%)	Validation (%)	Test (%)
Accuracy	99.43	84.96	84.32
Precision	99.77	83.66	83.25
Recall	99.08	87.56	84.87

**Figure 7:**
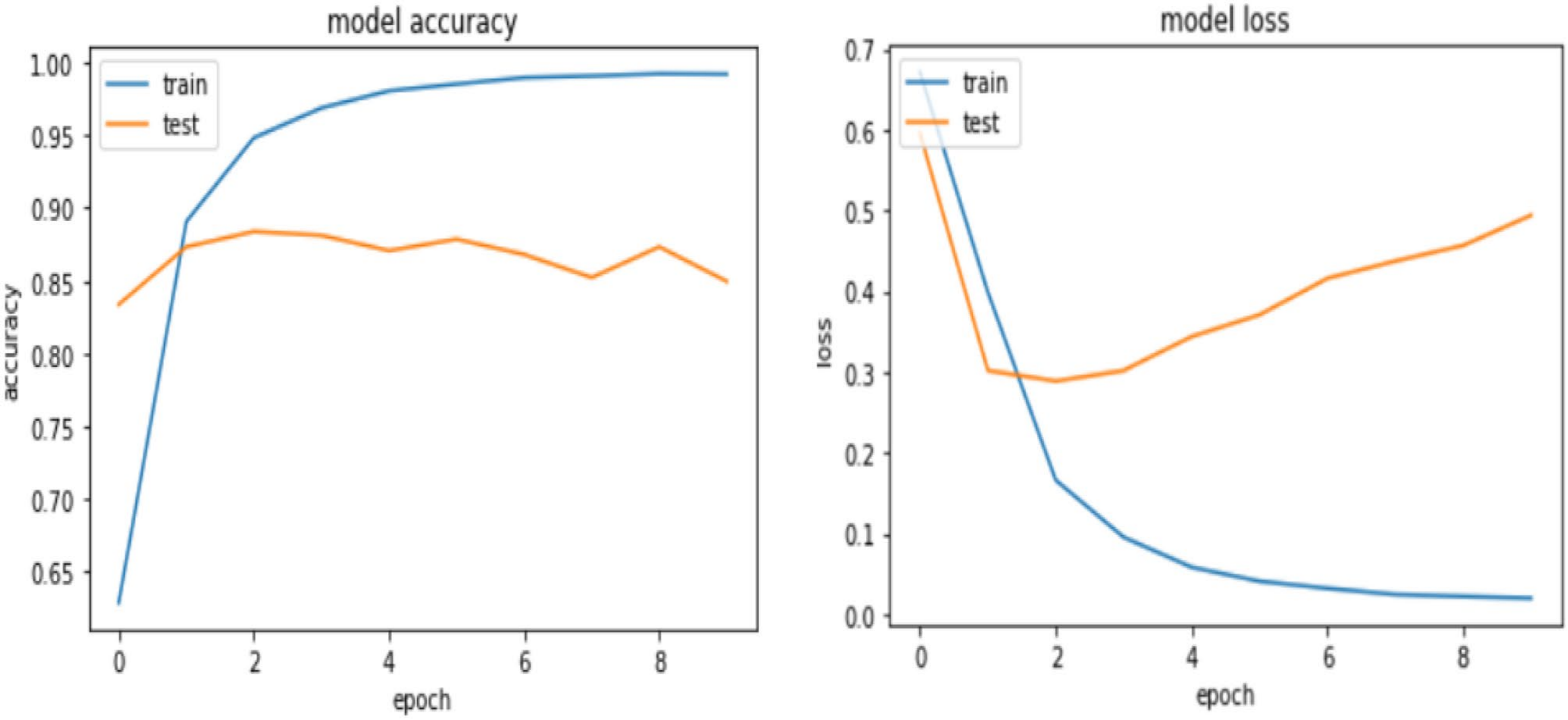
Learning curve for the CNN model.

From the above Table 4 it is observed that the model achieved 99.43% accuracy for the training dataset while it achieves 84.96 and 84.32 for validation and testing accuracy, respectively. The learning curve is depicted in Fig. 7 below.

The training accuracy increases as the number of epochs increases, but the Validation accuracy decreases as the number of epochs increases. As a result, it can be concluded that the model is over-fitted. When compared to the work required to combat over-fitting, building a model and executing the code is the easier part. The researcher used many regularization approaches for our model, such as Seeding (also known as Random state) from 42 to 50. To reduce the model’s vulnerability to over-fitting, the researcher added one Dense layer (Hidden layers) with 64 neurons and the activation function ReLU. Then added a dropout layer to the Convolutional layer before feeding it into the pooling layer, then added a dense layer. After the dense layer, the researcher also added another dropout layer, which was then fed into the fully connected layer. Dropout was discovered to be incredibly essential since it allows the model to avoid over-fitting by dropping neurons at a random point. The batch size was increased from 64 to 100, and the epoch number was decreased from 10 to 9. Change is made based on manual tunning and the experimental result is presented in Table 5.

**Table 5:** Model result after regularization.

Metrics	Training (%)	Validation (%)	Test (%)
Accuracy	89.63	87.11	84.79
Precision	79.62	80.32	80.39
Recall	72.81	73.57	73.69

**Figure 8:**
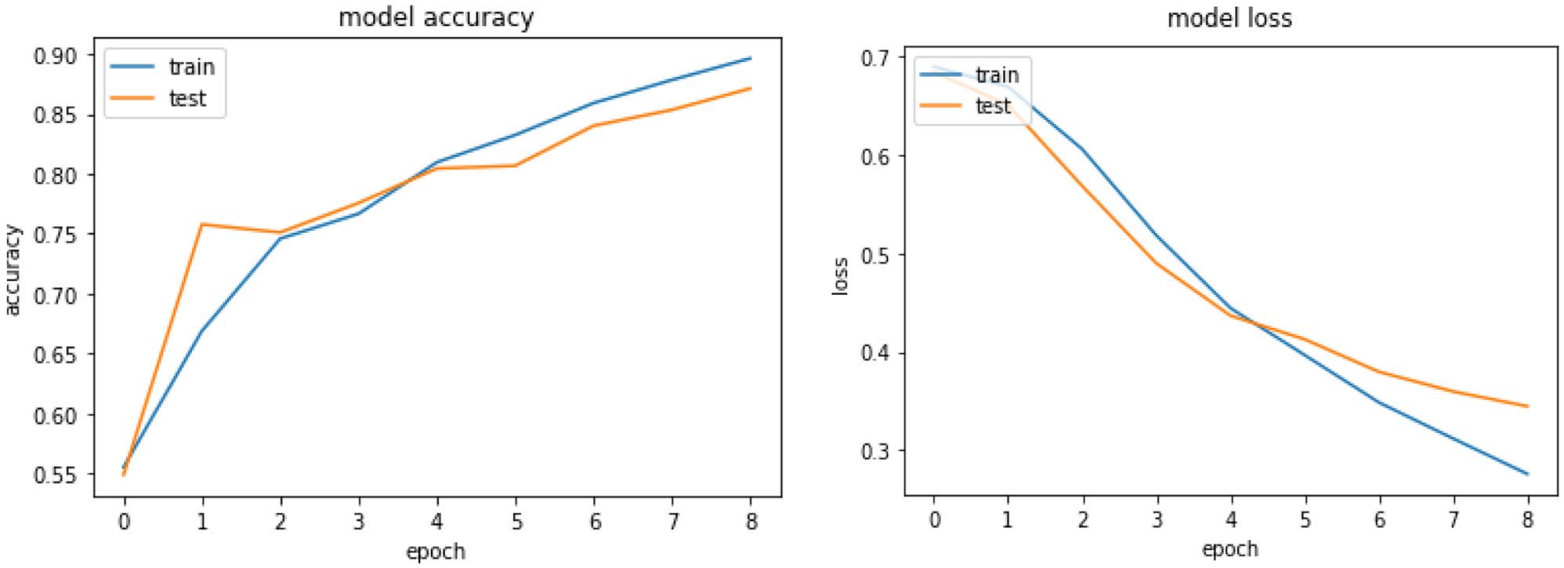
Learning curve for CNN model after regularization.

As presented in Table 5, after regularization, the accuracy of the model was improved, and the result shows that there is minimal difference observed among training, validation, and test accuracy. This further shows that the problem of over-fitting is solved as compared to the previous result achieved before regularization. Figure 8 also shows the learning curve of the CNN Model after regularization.

From the learning curve in Fig. 8, the model has no overfitting problem since the gap that was shown between the training and the validation has been decreased. The CNN model for Amharic sentiment dataset has finally registered an accuracy, Precision, recall of 84.79%, 80.39%, and 73.69% respectively.

### Experimenting using bidirectional-LSTM

The Bidirectional-LSTM layer receives the vector representation of the data as an input to learn features once the data has been preprocessed and the embedding component has been constructed. Bi-directional LSTM (Bi-LSTM) can extract important contextual data from both past and future time sequences. Bi-LSTM, in contrast to LSTM, contains forward and backward layers for conducting additional feature extractions which is suitable for Amharic language because the language by its nature needs context information to understand the sentence. Bi-LSTM has one hidden layer for each direction to extract features. One copy of the hidden layer fits in the input sequences as the traditional LSTM, while the other is placed on a reversed copy of the input sequence. The results obtained from all these LSTMs are concatenated by default. For both the forward and backward hidden layers in our model, the researcher used a bidirectional LSTM with a 64-memory unit. Then add a dropout of (0.4, 0.5), Random state of 50, Embedded size of 32, batch size of 100, and 3 epochs to minimize overfitting. To calculate the loss function Binary Classification were used and Adam as an optimizer. The experimental result of Bi-LSTM is presented in Table 6.

**Table 6:** Bi-LSTM model evaluation.

Metrics	Training (%)	Validation (%)	Test (%)
Accuracy	90.76	89.18	85.27
Precision	90.99	90.06	85.24
Recall	90.88	87.63	81.67

**Figure 9:**
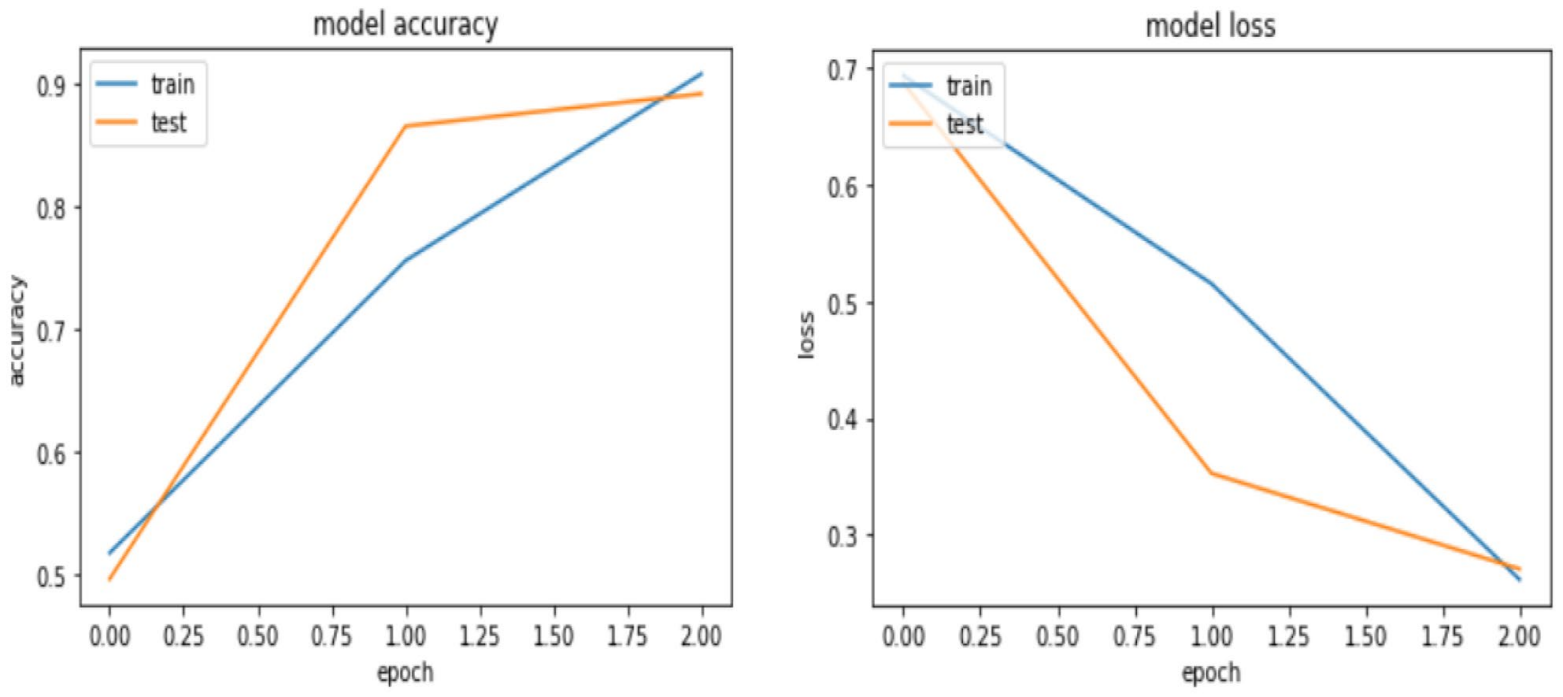
Learning curve for Bi-LSTM model.

The Bi-LSTM model result shows an accuracy of 90.76%, 89.18%, and 85.27% for the training, validation, and testing respectively. Hereunder Fig. 9 presents the learning curve of Bi-LSTM.

From the learning curve depicted in Fig. 9 that, the difference between the training and validation accuracy is nominal, indicating that it is not overfitted and hence capable of generalizing to previously unknown data in the real world. The model result shows a satisfactory fit to our dataset. To get to the ideal state for the model, the researcher employed regularization approaches like dropout as discussed above.

The accuracy, precision, and recall of the Bi-LSTM for Amharic sentiment dataset were 85.27 percent, 85.24%, and 81.67%, respectively. The result shows that BI-LSTM model performs better than CNN model which further indicates the capability of BI-LSTM to improve the classification performance by considering the previous and future words during learning.

### Experimenting using GRU

For GRU first, the researcher creates a suitable embedding layer with the maximum feature and provide the output shape. Between the embedding layer and the hidden layer, the input values serve as weights. Gated recurrent units make up the hidden layer. The researcher used GRU with two layers and get the representation of the entire sequence that was then passed as input to the outer layer, which used the Sigmoid activation function to categorize the sentiment as positive or negative and Adam as optimizer. For each GRU 64 units and 32 units of memory were used. After building the model, the test result shows the model was overfitted. So, to overcome overfitting the researcher added a dropout of (0.5, 0.5), change the Random state from 50 to 42, batch size of 128, and 10 epochs. The one hyperparameter that made the difference was modifying the default value of Adam learning rate from 0.1 to 0.0001. Table 7 below shows the experimental result of GRU.

**Table 7:** GRU model evaluation result.

Metrics	Training (%)	Validation (%)	Test (%)
Accuracy	97.73	92.67	88.99
Precision	89.89	90.63	90.61
Recall	88.82	89.65	89.67

**Figure 10:**
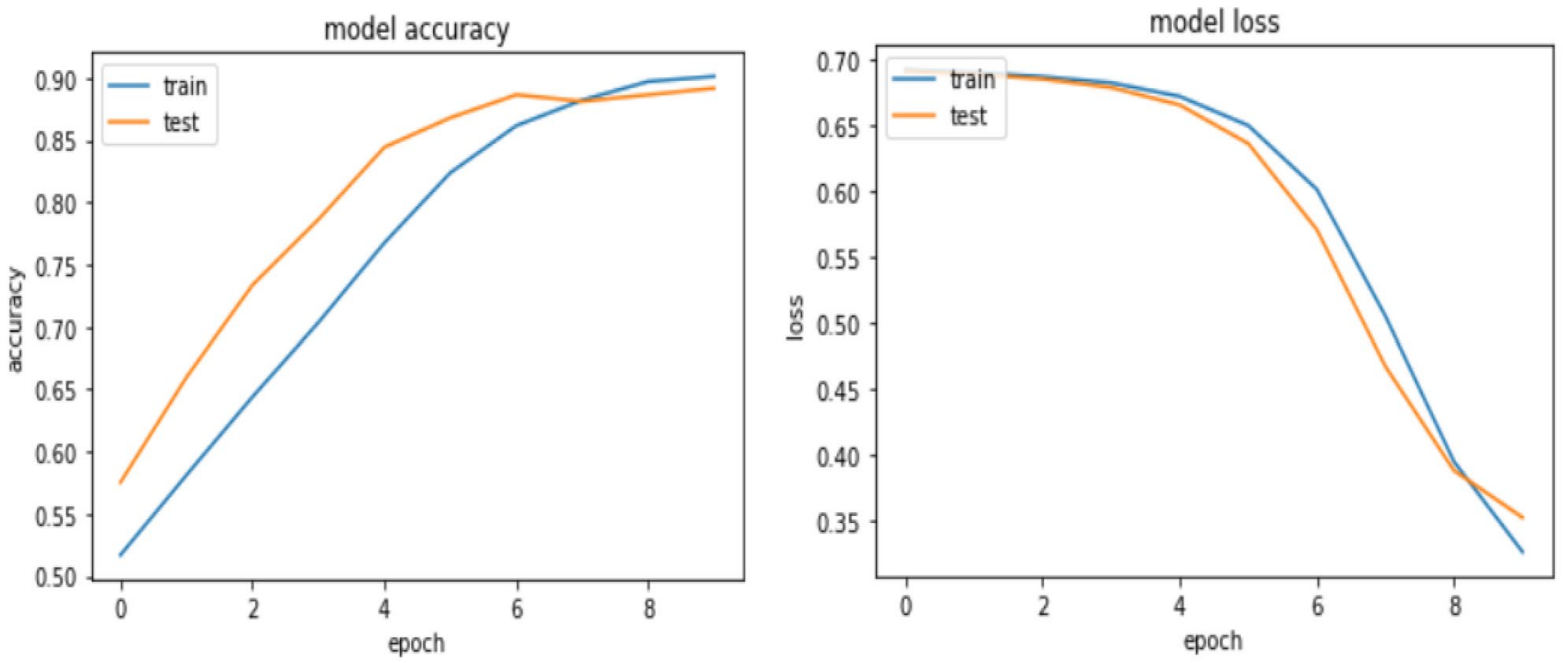
Learning curve for GRU model.

As presented in Table 7, the GRU model registers an accuracy of 97.73%, 92.67%, and 88.99% for the training, validation, and testing, which are close to the result that was obtained for BI-LSTM. Though the number of epochs considered for the GRU to get this accuracy is twice that of BI-LSTM, GRU solves the over-fitting challenge as compared to Bi-LSTM with some parameter tuning. Figure 10 depicts the learning curve of the GRU model.

From the learning curve of the GRU model, the gap between the training and the validation accuracy is minimal, but the model at the start begins to underfit. However, when the researcher increases the epoch number, the accuracy increased, which overcomes underfitting. The loss was high with 64% at the first iteration, but it decreases to a minimum in the last epoch to 32%. In the end, the GRU model converged to the solution faster with no large iterations to arrive at those optimal values. In summary, the GRU model for the Amharic sentiment dataset achieved 88.99%, 90.61%, 89.67% accuracy, precision, and recall, respectively.

### Experimenting using CNN-bidirectional-LSTM

When the researcher combined CNN and Bi-LSTM, the intention is to take advantage of the best features of each model to develop a model that could comprehend and classify the Amharic sentiment datasets with better accuracy. Combining the two models will provide the best feature extraction with context understanding. From the embedding layer, the input value is passed to the convolutional layer with a size of 64-filter and 3 kernel sizes, as well as with an activation function of ReLU. After the convolutional layer, there is a max-pooling 1D layer with a pool size of 4. The output from this layer is passed into the bidirectional layer with 64 units. The output was then passed into the fully connected layer with Sigmoid as the binary classifier. For the optimizer, Adam and Binary Cross entropy for loss function were used. The result is shown below in Table 8.

**Table 8:** CNN-Bi-LSTM model evaluation result.

Metrics	Training (%)	Validation (%)	Test (%)
Accuracy	99.63	88.13	87.88
Precision	99.50	87.53	87.43
Recall	99.50	84.38	87.86

**Figure 11:**
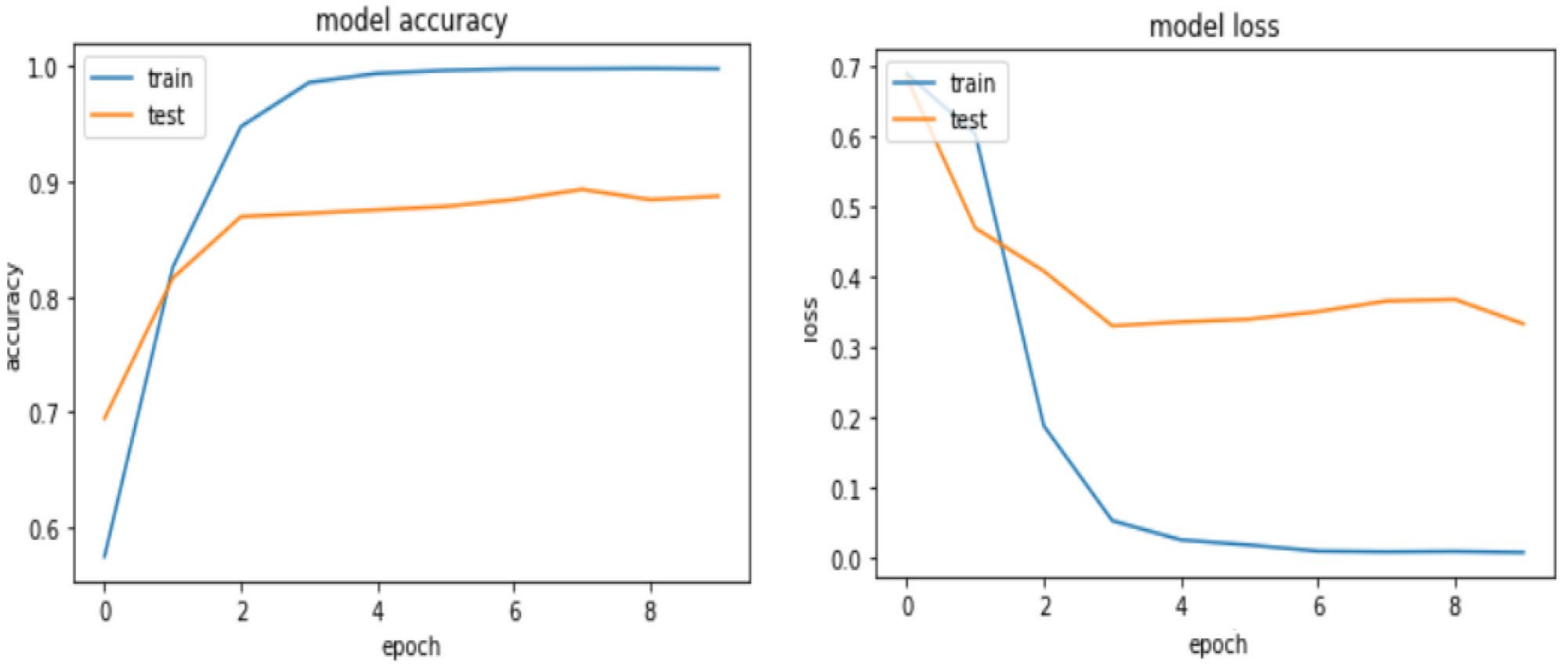
Learning curve for hybrid CNN and Bi-LSTM model.

From Table 8, the trained model registers accuracy, precision and recall of 99%, while the model performs poorly during validation and testing on the given unseen datasets. This shows the model is memorizing the training data instead of learning, which resulted in over-fitting. Below the learning curve depicted in Fig. 11 shows the behavior of model accuracy vs. model loss.

The Learning curve in Fig. 11 shows the training loss is close to 0 while the loss for the validation set is increasing which indicates overfitting. To overcome overfitting, the researcher applied different first regularization methods like weight decaying, adding dropouts, adjusting the learning, batch size, momentum of the model, and reducing the iteration of the model. Various hyperparameters were tuned until the model’s optimal value was reached, which shifted it from overfitting to an ideal fit for our dataset.

Table 9 shows the optimal values for CNN-BI-LSTM.

**Table 9:** Optimal value for tuning the CNN-BI-LSTM model.

Dropout	0.2 and 0.3
Learning rate	0.0001
Momentum	0.7
Epoch No	5
Batch size	128
random state	0

Using the aforementioned optimized hyperparameters depicted in Table 9, the experimental result is shown below in Table 10.

**Table 10:** Evaluation result CNN-Bi-LSTM model after hyperparameter tuning.

Metrics	Training (%)	Validation (%)	Test (%)
Accuracy	99.73	91.11	91.60
Precision	99.56	94.37	90.47
Recall	99.89	87.77	93.91

**Figure 12:**
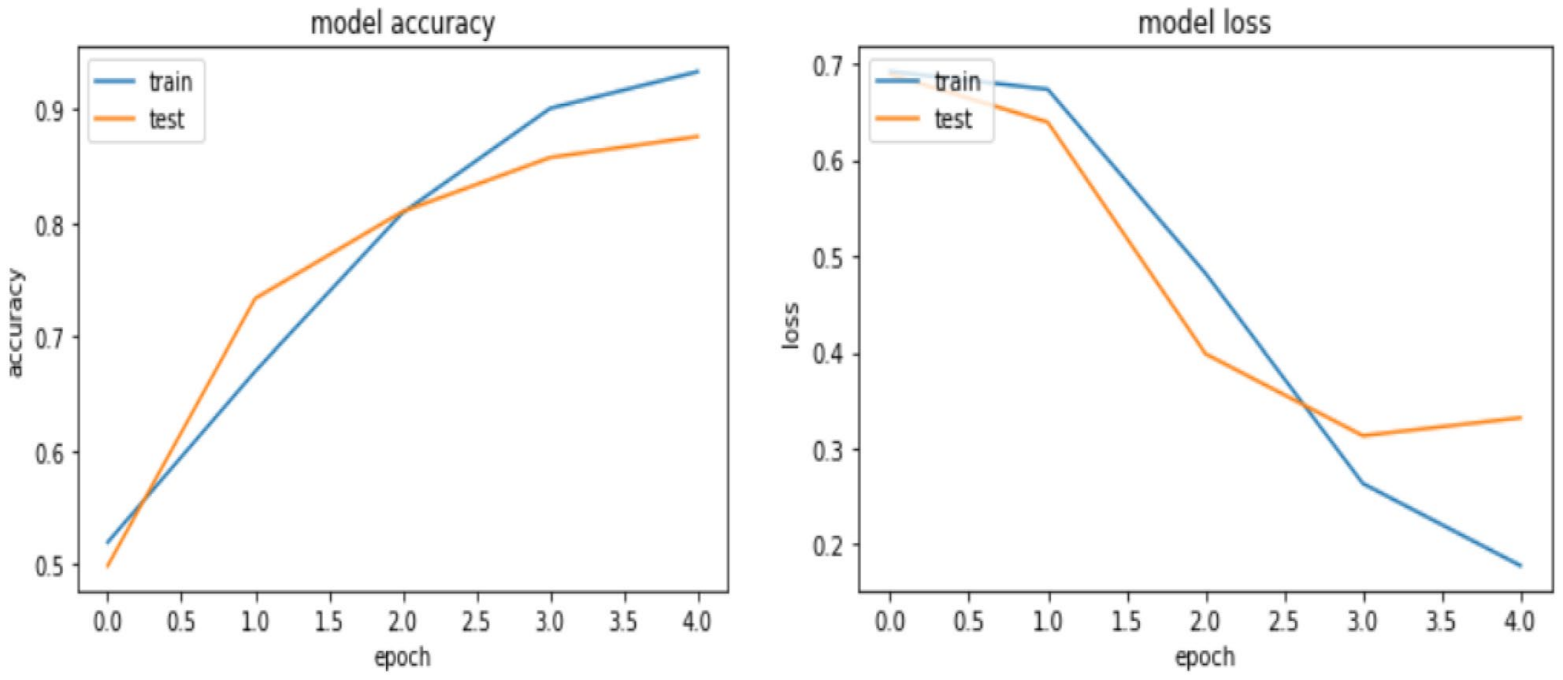
Learning curve for hybrid CNN and Bi-LSTM model.

As shown in Table 10, 99.73%, 91.11% percent, and 91.60% percent accuracy were achieved for training, validation, and testing, respectively. This hybrid model outperforms previous models, and when looking at the marginal differences between training, validation, and testing, the difference is small, showing how well the model works in unknown datasets and its generalization ability. Figure 12 depicts the learning curve of the hybrid CNN and Bi-LSTM model.

Overall, for the Amharic sentiment dataset, the CNN-Bi-LSTM model achieved 91.60%, 90.47%, 93.91% accuracy, precision, and recall, respectively.

## Comparison of models

**Figure 13:**
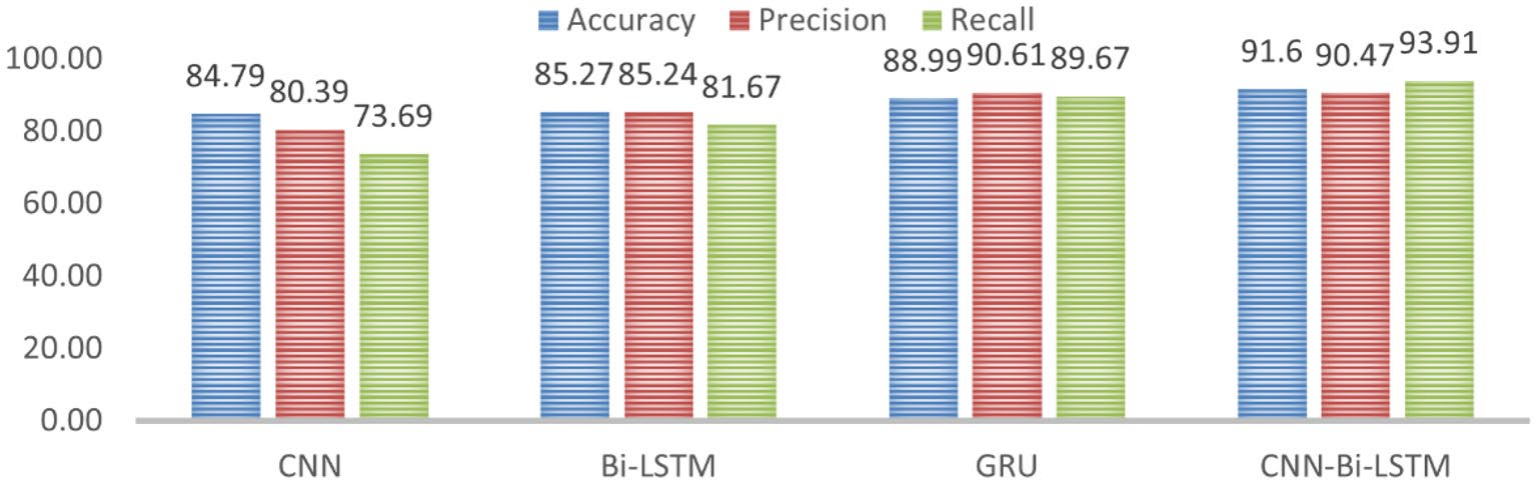
Comparison of models.

The experiments were performed using four distinct deep learning models, based on which promising results for Amharic sentiment analysis were obtained. Figure 13 presents the comparison between the four models.

**Figure 14:**
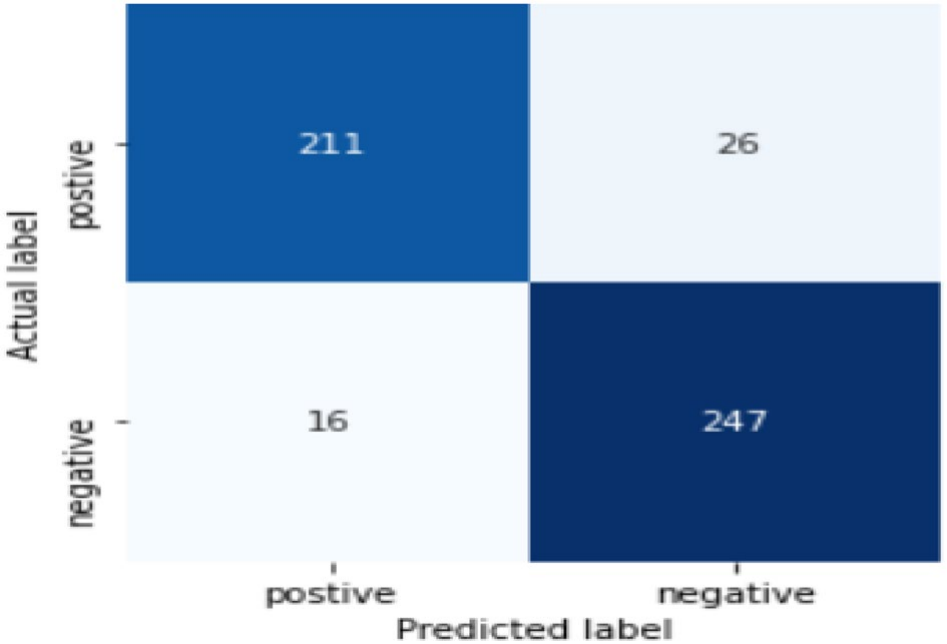
Confusion matrix for CNN-Bi-LSTM.

Figure 13 shows, the performance of the four models for Amharic sentiment dataset, and when comparing their performance CNN-BI-LSTM showed a much better accuracy, precision, and recall. CNN-Bi-LSTM uses the capability of both models to classify the dataset, which is CNN that is well recognized for feature selection, while Bi-LSTM enables the model to include the context by providing past and future sequences. Combining these two models, the accuracy was 91.60%. Figure 14 provides the confusion matrix for CNN-BI-LSTM, each entry in a confusion matrix denotes the number of predictions made by the model where it classified the classes correctly or incorrectly. Out of the 500-testing dataset available for testing, CNN-BI-LSTM correctly predicted 458 of the sentiment sentences. The Misclassification Rate is also known as Classification Error shows the fraction of predictions that were incorrect. It is calculated using the following equation.

$$\mathrm{Misclassification\, rate }=\frac{FP+FN}{TP+TN+FP+FN}$$

The misclassification rate for CNN-BI-LSTM is calculated first by adding false positive and false negative, divided by the total testing dataset. False positive for this model is 26, while the False negative is 16, which gives a misclassification rate of 8.4% for the model, which showed a low misclassification rate. The confusion matrix in Fig. 14 shows that the number of false-positive are higher than that of false negative. Table 11 shows type one and type two errors encountered by the model.

**Table 11:**
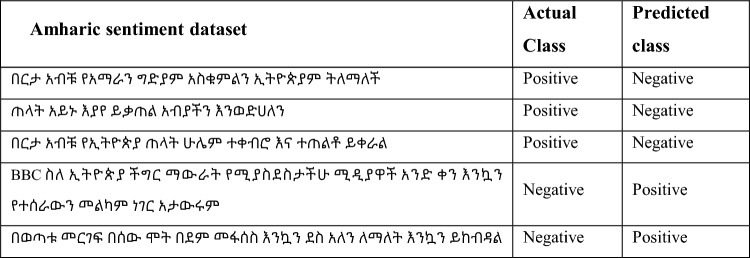
Examples of misclassification by the model.

Table 11 show that the model gets confused when it found comments that have sarcasm, figurative speech, or sentiment sentences that contain both words that give positive and negative sentiment in one comment. For example,  and  the first sentence contains the positive words like  while the second sentience contain . But it also contains words that imply a negative sentiment like for the first sentence  while the second contains  the above sentences belong to a positive class, but the model predicted it as negative because of the words contained within the sentence which caused misclassification.  the word  implies a positive sentiment while the overall sentiment of the comment is negative caused the model to predict the sentiment as positive. From the CNN-Bi-LSTM model classification error, the model struggles to understand sarcasm, figurative speech, mixed sentiments that are available within the dataset.

## Discussion of results

This research addresses gaps from previous works through a comprehensive experimental study. The researcher studied the impacts of datasets preparation, word embedding, and deep learning models, with a focus on the problem of sentiment analysis. Four deep learning models CNN, Bi-LSTM, GRU, and CNN-Bi-LSTM for Amharic sentiment analysis were compared, the experiment result showed that combining CNN with Bi-LSTM generated a model that outperformed the others. Each model was compared at the model’s specific optimal point; that is, when the models reached their good fit. CNN-Bi-LSTM takes advantage of the strengths of the two models; CNN is recognized for its ability to extract as many features as possible from a sentence and Bi-LSTM keeps the chronological order between words from past and future which enables the model to understand context.

Several factors influence the performance of deep learning models for instance data preparation, the size of the dataset, as well as the number of words within the sentence impact the performance of the model. When training the model using 3000 sentences of the datasets and with a limited number of words within a sentence gives an accuracy of 85.00%. As the number of words increases to greater than five words per comment within the sentence the performance improves from 85.00 to 88.66% which is a 3.6% improvement. Whereas increasing the size of the dataset to 5000 showed an accuracy of 91.60 which is a 3% upgrade. From the results, we can see the impact the size of the dataset, as well as the size of words within a single comment, has on the performance of the model. Other factors like word embedding, filters size, kernel size, pool size, activation function, batch size, adjusting hyperparameter and the optimization mechanism also play a major role in the performance of the models. Overall tuning the above factors showed a significant amount of improvement to the deep learning model performance. But factor such as padding respond differently from model to model for instance applying pre-padding to CNN increases the model performance by 4% while other models perform poorly using pre-padding.

Kapočiūtė-Dzikienė et al.^29^, claim that deep learning models tend to underperform when used for morphologically rich languages and hence recommend traditional machine learning approach with manual feature engineering. Despite the author’s conclusion, the recommendation does not hold true when comparing the performance of Amharic sentiment analysis model constructed in this study using deep learning with machine learning model proposed by Refs.^6, 18^. Findings from this study show deep learning models bring improvement compared to traditional machine learning in terms of work needed for feature extraction, performance, and scalability. Manual feature engineering wasn’t used for this work; so, it eliminates extra effort that was needed for feature extraction and in addition, the models could understand the context of a given sentence. When considering the model’s performance, a small (+ 1%) but significant increase was achieved. Scalability is the main challenge for standard machine learning models while the deep learning models used in this research showed that the accuracy for the model increases as the size of the dataset for training and testing increases.

Two researchers attempted to design a deep learning model for Amharic sentiment analysis. The CNN model designed by Alemu and Getachew^8^ was overfitted and did not generalize well from training data to unseen data. This problem was solved in this research by adjusting the hyperparameter of the model and shift the model from overfitted to fit that can generalize well to unseen data. The CNN-Bi-LSTM model designed in this study outperforms the work of Fikre^19^ LSTM model with a 5% increase in performance. This work has a major contribution to update the state-of-the-art Amharic sentiment analysis with improved performance.

The proposed model achieved 91.60% which is 6.81%, 6.33%, and 2.61% improvement from CNN, Bi-LSTM, and GRU respectively. The proposed model achieved a very promising result for sentiment analysis. Mostly in this research work, overfitting was encountered but different hyperparameters were applied to control the learning process. Hyperparameters like Learning rate, dropout, Momentum, and random state for our case shifted the model from overfitting to a good fit. If a model achieved a high accuracy but is overfitted it won’t be useful in the real world because the model generalization capacity is not applicable.

## Conclusion

In Ethiopia, a lot of opinions are available on various social media sites, which must be gathered and analyzed to assess the general public’s opinion. Finding and monitoring comments, as well as extracting the information contained in them manually, is a tough undertaking due to the huge range of opinions on the internet. As a matter of fact, the normal human reader will have trouble finding appropriate websites, accessing, and summarizing the information contained inside. As a result, automated sentiment analysis methods are necessary. Different researchers used sentimental analysis for Amharic sentiment either with Lexical or Machine Learning. Both approaches require the interference of the programmer at one point or another. But when it comes to deep learning it minimizes human involvement which makes life easier. In this research, the researcher applied sentimental analysis on Amharic political sentences using four different deep learning approaches; CNN, Bi-LSTM, GRU, and hybrid of CNN with Bi-LSTM. To the researcher’s knowledge, this is the first work that applied BI-LSTM, GRU, and CNN-Bi-LSTM.

Experimental result shows that the hybrid CNN-Bi-LSTM model achieved a better performance of 91.60% compared to other models where 84.79%, 85.27%, and 88.99% for CNN, Bi-LSTM, and GRU respectively. The researcher conducts a hyperparameter search to find appropriate values to solve overfitting problems of our models. While these results verify the main contribution of the study there is still room for improvement. When working on this research problems like manually collecting and annotating the dataset is a very tiring task. Even though a promising accuracy was achieved the model was trained with limited dataset which made the model learn only limited features and only considered binary classification. The model struggle to distinguish sarcasm, figurative speech and sentiment sentences that contain both words that give positive and negative sentiment. These challenges are area that need further research.

## Recommendation

This research underscores the significance of adopting a multi-class classification approach over the conventional binary positive–negative scheme. Because a multi-class framework offers a more nuanced and insightful breakdown of sentiments. Furthermore, the establishment of a standardized corpus emerges as a crucial endeavor. While this study’s primary focus revolves around political sentiment analysis, its applicability extends far beyond the political domain. The insights and methodologies developed herein can be readily extended to diverse sectors such as agriculture, industry, tourism, sports, entertainment, and areas concerning both employee and customer satisfaction. In the future research, a notably unexplored avenue pertains to the analysis of sarcastic comments in the Amharic language, presenting a promising area for further investigation.

## Data Availability

The datasets used and/or analyzed during the current study available from the corresponding author on reasonable request.
